# Peer Mindset Culture as a Developmental Context for Belonging

**DOI:** 10.1007/s10648-025-10082-8

**Published:** 2025-10-30

**Authors:** Eunjin Seo, Margarett Clapper, Cameron A. Hecht, Robert Crosnoe, David S. Yeager

**Affiliations:** 1 School of Family and Consumer Sciences, Texas State University, San Marcos, TX USA; 2 The Univeresity of Texas at Austin, Austin, TX USA; 3 University of Rochester, Rochester, NY USA

**Keywords:** Peer, Mindset, Belonging, Culture, Adolescent

## Abstract

Over the past decade, mindset research has increasingly moved beyond the individual, examining how beliefs about ability are shaped and reinforced by the broader social context. This paper extends that work by focusing on peers, a powerful but underexplored influence in adolescent development. We introduce a theoretical framework for understanding peer mindset culture: the shared beliefs that peers hold and communicate about whether ability is fixed or can grow. We argue that fixed mindset peer cultures can erode students’ sense of belonging by (a) triggering identity threats (e.g., stereotype threat, perceived mismatch with peers’ beliefs, or social contagion of fixed norms), and (b) weakening social connection (e.g., peer selection and low support). We argue that these dynamics are especially consequential for students from minoritized backgrounds, those who are struggling academically, and those who personally endorse a growth mindset. Finally, we outline principles for designing peer-focused mindset interventions. Our goal is to provide avenues for future research and practical strategies to support all students in developing a strong sense of belonging in their learning environments.

## Introduction

When students transition into new academic settings, they often grapple with two questions: Can I succeed here? Do I belong? These questions are not just parallel concerns; they are psychologically intertwined, particularly during adolescence and early adulthood, when young people are actively forming their academic identities, and those identities are often shaped by their social standing among peers (Albert et al., 2013; Yeager, 2024, Chapter 9). Research on mindsets has made clear that students’ beliefs about ability are important for shaping their sense of competence and resilience (Burnette et al., 2013; Dweck & Yeager, 2019; Seo et al., 2019). And decades of work on belonging show that sense of belonging—students’ perceived fit in a context (Walton & Brady, 2017)—predicts a wide range of outcomes (Allen et al., 2021; Fong et al., 2024; van Kessel et al., 2025). Yet this literature often moves in parallel rather than in conversation. Importantly, one underexplored point of intersection is peer mindset culture—peers’ shared beliefs about whether ability is fixed or can grow—and how it shapes students’ sense of belonging. This paper proposes a new framework that centers peer mindset culture as a contextual influence on belonging and maps the psychological mechanisms through which it may operate.

## Rethinking Mindset: From Individuals to Contexts

Classic mindset theory (Dweck & Leggett, 1988; Dweck et al., 1995) posits that when students believe ability is fixed, they are more likely to disengage in the face of difficulty, avoiding challenges and giving up quickly. In contrast, students who believe ability can grow tend to adopt a mastery orientation, embracing effort and persisting through setbacks. While this foundational work focused on individual beliefs, it opened the door to a broader question: What shapes those beliefs in the first place?

In recent years, researchers have begun to explore how context can send messages about whether ability is fixed or malleable (Canning & Limeri, 2023; Seo et al., 2025c). These contextual cues matter. For example, the field-specific ability beliefs hypothesis suggests that when a field is perceived as requiring brilliance rather than effort or dedication for success, students from minoritized backgrounds may feel like they do not belong, particularly in domains like mathematics, physics, or philosophy (Bauer et al., 2025; Leslie et al., 2015; Meyer et al., 2015; Muradoglu et al., 2022). That perception alone can drive students away.

This shift toward context has been formalized in the Mindset × Context perspective (Hecht et al., 2021; Walton & Yeager, 2020; Yeager & Dweck, 2020). The key insight: Growth mindset beliefs are most impactful when the environment supports and reinforces them. If a student believes they can grow but their environment tells them otherwise, through subtle norms, messages, or behaviors, their motivation and sense of belonging may still falter. Much of the research in this space has focused on teachers and institutions (e.g., Hecht et al., 2023a, b; Kroeper et al., 2022a, b; Murphy & Reeves, 2019), but peers play a uniquely powerful role, especially during adolescence (Albert et al., 2013; Yeager et al., 2017, 2018). Although a few studies have begun to examine peer mindset cues (e.g., Muenks et al., 2021; Seo et al., 2025c; Sheffler & Cheung, 2020), the field lacks a coherent framework to explain *how* peer mindset cultures influence students’ sense of belonging.

Our contribution is to zoom in on this question. We propose that peer mindset cultures are a critical, yet underexamined, context for understanding adolescent belonging. This focus allows us to unpack the specific mechanisms through which peer environments may support or undermine students’ sense of belonging. While our primary focus is on mindsets about intelligence and academic ability, we also acknowledge that students hold domain-specific mindset beliefs across a range of traits, such as creativity, leadership, or personality (e.g., Chiu et al., 1997; Kangas et al., 2023; Karwowski, 2014). In environments where those traits are emphasized, we believe that fixed beliefs in those domains can similarly shape whether students feel seen, valued, and able to succeed.

We draw on and extend multiple theoretical perspectives. The Integrative Framework of Belonging (Allen et al., 2021) and the Many Questions of Belonging framework (Walton & Brady, 2017) help clarify what it means to belong and how peer mindset cultures may shape that experience. We also borrow insights from Motivational Climate Theory (Robinson, 2023) to articulate how observable peer behaviors (e.g., encouragement, feedback, challenge-seeking) create microclimates that influence students’ sense of belonging. By bringing these strands together, our framework aims to move the field toward a more precise, developmentally informed understanding of how peer culture shapes belonging and how we might intervene to improve them.

## Peers as Primary Socializers in Adolescence

Much of what we know about shaping students’ mindset comes from research on parents and teachers (King, 2020). That makes sense: adults structure students’ environments, set expectations, and model beliefs. Parents lay the groundwork in early childhood, offering emotional security and foundational beliefs about learning and self-worth (e.g., Sanders & Turner, 2018; Seo et al., 2025d; van IJzendoorn, 1995). Teachers play a critical role in creating classroom norms that promote growth and inclusion (e.g., Jimerson & Haddock, 2015; Wang et al., 2020). 

But in adolescence and early adulthood, the center of gravity begins to shift. Young people spend more time with peers than with adults (Larson & Richards, 1991), and peer relationships become increasingly central to how they see themselves and where they fit (Reitz et al., 2014; Yeager et al., 2018). This is a developmental turning point. Adolescents become highly attuned to peer norms and status hierarchies (Murphy et al., 2013; Spear, 2000), and their experiences of belonging or exclusion are increasingly shaped by their interactions with peers (Craggs & Kelly, 2018; Drolet & Arcand, 2013). When asked what makes them feel like they belong in school, adolescents consistently point to their peers: being accepted, having close friendships, and feeling that others want them around (Drolet & Arcand, 2013). Peer relationships often outweigh parental or teacher support during this period, especially when it comes to navigating academic challenges and mental health concerns (Bokhorst et al., 2010; Wecht et al., 2023). And because most peer relationships are reciprocal and emotionally salient (Reitz et al., 2014), peers are not just bystanders; they are powerful co-creators of cultures (Seo et al., 2025c).

How do peers shape each other’s beliefs? One way is through direct socialization. During adolescence, peer socialization often outweighs the influence of parents or teachers (Verschueren et al., 2012). Most adolescents maintain reciprocal friendships (Reitz et al., 2014) and often find peers to be a more significant source of support than parents or teachers (Bokhorst et al., 2010). Consequently, they frequently turn to peers for discussions on important issues, such as mental health, rather than seeking guidance from parents (Wecht et al., 2023). This highlights the potential importance of peers in shaping mindset beliefs; even if a youth starts with a growth mindset from their parents, this mindset can shift after interacting with peers who hold a fixed mindset (Fong et al., 2025b; King, 2020), affecting their sense of belonging (Muenks et al., 2021; Seo et al., 2025b, c).

Peers also act as amplifiers. They repeat, reshape, and sometimes reinterpret the messages adults try to send (Crosnoe & Muller, 2014; Radel et al., 2010). When a teacher encourages persistence, peers might echo that message or undermine it through social cues or sarcasm. In this way, peers function as a kind of “social filter” for adult messaging. At times, they also reinterpret and innovate. Youth-led social movements, from climate activism to racial justice to expanding acceptance of gender fluidity, often emerge when young people perceive adult institutions as too slow or rigid (e.g., Liou & Literat, 2020; Twenge, 2023). These shifts in peer norms highlight that peers do not just receive cultural messages; they can lead cultural change.

For this reason, we think that interventions aimed at improving belonging must take peer dynamics seriously. It is not enough to target individual beliefs or adult behaviors; we also need to understand and influence the peer cultures that shape students’ daily experiences. Our framework begins to address this by asking: How do peer mindset cultures shape students’ sense of belonging, and how can we support students in creating growth-oriented peer cultures?

## How Peer Mindset Cultures Might Influence Belonging: Two Theoretical Pathways

An emerging body of empirical studies (e.g., Muenks et al., 2021; Seo et al., 2025b) along with a recent review (Seo et al., 2025c) indicate that peer mindset cultures play a critical role in shaping students’ sense of belonging. Yet, we lack a clear framework explaining *how* these cultures exert their influence. Without that explanatory logic, it remains difficult to determine whether peer mindset culture is a causal driver of belonging, or whether both are shaped by some third factor. More importantly, without identifying the specific pathways at play, we are left with a blunt tool: we know peer norms matter, but we do not know how to change them or for whom change would be most impactful (Nielsen et al., 2018; Sumner et al., 2018). This matters, especially because a sense of belonging is a foundational ingredient in students’ educational trajectories, particularly for students from minoritized groups who face frequent signals that they do not fit (Fisher et al., 2020; Fong et al., 2024; Walton & Cohen, 2007). To build more effective interventions, we need to move from asking if peer mindset cultures shape belonging to understanding how they do so and under what conditions.

**Fig. 1:**
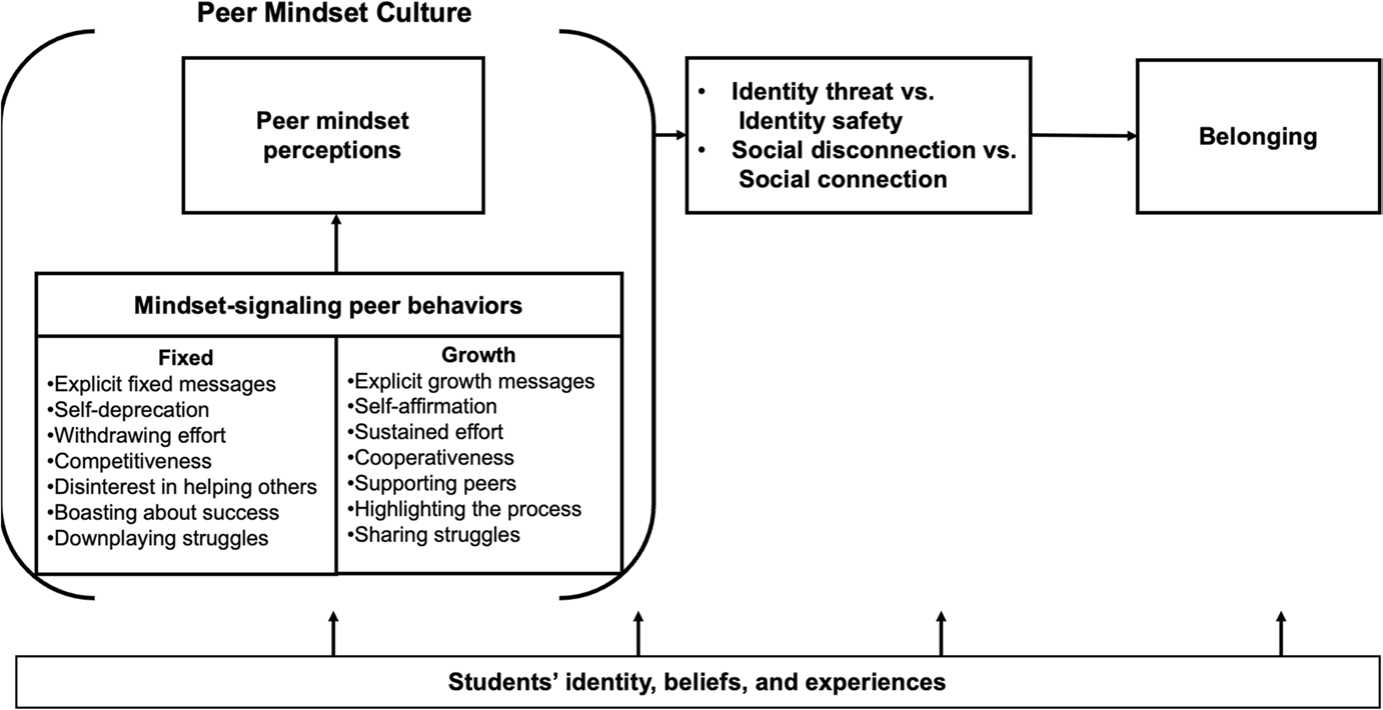
The proposed mediation model of peer mindset culture and belonging

Although no existing framework directly maps this terrain, we draw on two prominent perspectives on belonging—the Many Questions of Belonging framework (Walton & Brady, 2017) and the Integrative Framework of Belonging (Allen et al., 2021)—to propose two broad pathways through which fixed mindset peer cultures can erode belonging (Fig. 1). The first pathway is identity threat. Students may come to see their valued identities as misaligned with the implicit norms and values of their environment. This is not simply a matter of being excluded; it is the perception that “people like me” are not seen as capable, accepted, or able to succeed here. That can powerfully undermine one’s motivation to belong, especially for students from groups that have historically been minoritized in academic settings (Kroeper et al., 2025; Lee et al., 2025).

The second pathway involves reduced opportunities for connection. Belonging is not just about motivation; it also requires opportunities (Allen et al., 2021). Students need access to meaningful peer interactions in which they feel seen, valued, and supported. But fixed mindset peer cultures can narrow those opportunities (Freeman & Getty, 2023; Won & Chang, 2024). When norms discourage vulnerability, frame struggle as weakness, or pit students against one another, it may become harder to form authentic academic relationships. The result is a climate where even students who want to belong may find themselves isolated.

We propose that these two psychological pathways—identity threat and reduced opportunities for connection—can be further understood through five specific and empirically testable processes. Specifically, we theorize that stereotype threat, mindset-culture mismatch, and social contagion may function as mechanisms related to identity threat, while social isolation and low peer support may help explain how peer cultures shape students’ opportunities to connect (see Table 1 for definitions). These five processes, which we elaborate on in the following section, are not intended to be exhaustive or mutually exclusive. Rather, they are proposed as illustrative, testable pathways through which peer mindset culture might influence students’ everyday experiences of belonging.

**Table 1:** The definitions of the proposed mechanisms and their applicability

Mechanism	Definition	Applicability
**Identity threat**		
Stereotype threat	Situational predicament where individuals from minoritized groups experience anxiety and reduced performance in situations where they are aware of negative stereotypes about their grou’'s abilities	Students with minoritized identities
Mindset-culture mismatch	The incongruence between an individual’s cultural values and the dominant cultural values of their environment	Students with a strong growth mindset
Social contagion	The process by which behaviors, attitudes, and emotions spread through a group of people	Students with a weak or uncertain growth mindset
**Social disconnection**		
Peer selection	The process by which individuals choose friends or associates based on shared characteristics, such as beliefs, values, and personality traits	Students with a strong growth mindset and those from minoritized backgrounds
Low social support	Various forms of assistance, both emotional and practical, that individuals receive from their social network, including peers	Students who are academically struggling and those from minoritized backgrounds

It is also important for us to acknowledge that these mechanisms do not function identically for all students. Depending on a student’s background, developmental history, and current context, certain pathways may become more or less salient (Table 1). For example, stereotype threat may be an impactful experience for students with minoritized identities, whereas students without those identities but who struggle academically may be more affected by low social support. We also argue that these mechanisms are often interrelated and mutually reinforcing, creating compounding effects that deepen the impact of fixed mindset peer norms on students’ experiences of belonging. Figure 1 and Table 1 outline these processes and highlights how they may vary across student populations.

### Pathway 1: Belonging Undermined by Identity Threat

In this section, we explore how fixed mindset peer cultures may undermine belonging through identity threat pathways. Feeling that one’s important identities or core beliefs are respected is often essential to experiencing a sense of belonging (Browman, 2025; Walton & Brady, 2017). We draw on emerging evidence and theoretical arguments to examine how peers’ shared beliefs about ability can shape which social identities become salient, especially those linked to negative stereotypes. These beliefs can also expose mismatches between a student’s values and those of their peers or, over time, even pressure students to shift their core beliefs in order to fit in. Together, we argue that these dynamics can make students feel that their identities or values are not fully accepted in the learning environment, undermining their motivation to belong.

#### Example 1: Stereotype Threat

Stereotype threat refers to the psychological phenomenon where individuals from minoritized groups experience anxiety and reduced performance in situations where they are aware of negative stereotypes about their group’s abilities (Steele, 1997). In school settings, for example, this can manifest as the fear of confirming stereotypes about girls’ mathematical ability (Berdahl & Min, 2012; Spencer et al., 1999; Steele & Aronson, 1995).

We propose that fixed mindset peer cultures may act as a social amplifier of stereotype threat. When students are surrounded by peers who believe that ability is fixed and unchangeable, the social environment may begin to feel like a place where students are either naturally capable or not, leaving little room for growth, development, or second chances. This can make the stakes of confirming a stereotype feel more real (e.g., Aronson et al., 2002; Seo & Lee, 2021).

This idea is inspired by research on field-specific ability beliefs and brilliance stereotypes (Bauer et al., 2025; Bian et al., 2018; Leslie et al., 2015), which shows that the more a discipline is believed to require raw talent, the less diverse it becomes. While the Brilliance-Belonging Model (Bauer et al., 2025) and earlier pioneering work (Leslie et al., 2015) focuses on academic fields, we believe the same logic may apply at the peer group level. For example, a girl in a fixed-mindset peer group may become more self-conscious about the stereotype that girls are not “naturally” good at mathematics. But in a peer culture that embraces growth, effort, and improvement, those same stereotypes may feel less psychologically relevant or threatening. Although this mechanism has not yet been tested directly at the peer level, adjacent research provides early support. For instance, teacher’s fixed mindset has been linked to heightened stereotype threat and reduced interest (Seo & Lee, 2021; Seo et al., 2025a), and exposure to significant others who hold fixed beliefs has been associated with greater self-doubt (Reich & Arkin, 2006). This heightened awareness of the stereotype and self-doubt can eventually lead to a lowered sense of belonging (Good et al., 2012). In theory, peers may function similarly, or even more powerfully, because their approval and acceptance are so central during adolescence and early adulthood (Albert et al., 2013; Yeager, 2024; Yeager et al., 2017, 2018).

This process is likely to be most salient in contexts where group stereotypes are relevant and where students’ social identities are made salient by the structure of the setting. For example, in the U.S., East Asian students are often subject to stereotypes that portray them as competent but lacking in creativity or innovation (Lu, 2024). In a peer culture that values creativity and treats it as innate (Karwowski, 2014; Karwowski et al., 2020), East Asian students may experience stereotype-based identity threat even if they are academically successful. Similarly, in fields like mathematics or philosophy, where brilliance is often emphasized, women may face heightened identity threat due to cultural stereotypes that associate intellectual giftedness with men (Cimpian & Leslie, 2017).

Of course, not all students will be equally affected. For students whose identities are rarely targeted by negative stereotypes in a given setting, such as White boys in mathematics classrooms, this specific mechanism may be less relevant. However, that does not mean they are immune to the broader effects of peer mindset culture. As we explore in later sections, other pathways, such as low social support, may still influence their sense of belonging.

#### Example 2: Mindset-Culture Mismatch

A growing body of research on person–environment fit suggests that people feel a stronger sense of belonging when the values and norms of their environment align with their own (Higgins, 2000; Van Vianen, 2018). When that alignment is missing, such as when individuals perceive a mismatch between their beliefs and those of the group, feelings of alienation can follow (Browman, 2025). While this concept has traditionally been studied in terms of broad cultural values like independence vs. interdependence (Phillips et al., 2020; Stephens et al., 2012), we propose that a similar form of mismatch can occur at the level of mindset culture.

We propose the concept of mindset-culture mismatch to describe the experience of students whose beliefs about learning and ability are misaligned with the prevailing mindset norms in their surrounding environment, such as peers. We propose that students who strongly endorse a growth mindset, who see ability as something that can be developed through effort and learning, may find it difficult to feel at home in a peer environment that subtly or overtly promotes fixed ability beliefs. This is likely not because others are overtly hostile, but because the everyday cues in the environment may fail to affirm students’ values or support the kinds of learning behaviors they view as important.

Consider a student who views setbacks as part of the learning process. If they are surrounded by classmates who make self-deprecating comments when they fail, who avoid hard tasks to preserve a sense of being “smart,” or who boast about effortless success without struggles, that student may begin to feel out of place (Muenks & Yan, 2024; Seo et al., 2025b). The mismatch is not just about different learning strategies; it is about fundamentally different worldviews (Dweck & Yeager, 2019). As a result, students with a growth mindset may feel socially and psychologically disconnected, even when no overt stereotype is at play (Muenks et al., 2021; Seo et al., 2025b).

Emerging evidence supports this hypothesis. Canning et al. (2020) found that organizational growth mindset norms can foster a stronger sense of belonging, especially for individuals who already endorse a growth mindset. Similarly, Wallace et al. (2023) showed that students are more likely to feel drawn to and supported by institutions that match their own beliefs about learning and ability. And in some cases, endorsing a growth mindset in a fixed mindset culture may even backfire, leading to reduced well-being (Lou & Li, 2023).

Unlike stereotype threat, which disproportionately affects students with minoritized identities, we argue that mindset-culture mismatch may be most impactful for students who hold strong growth mindset beliefs. In this sense, the mechanism we propose here helps explain how even well-resourced or high-achieving students can experience low belonging when their peer culture conflicts with their values. Although this process may operate alongside other mechanisms, such as social contagion or low social support, it is unique in that it centers on an internal conflict between students’ core beliefs and the social cues around them.

#### Example 3: Social Contagion

Young people do not just learn from teachers or parents; they learn from each other (Harris, 1995; Ryan, 2000). Social contagion refers to the process by which emotions, behaviors, and beliefs spread through peer networks. Whether intentional or not, peers function as powerful socializers, shaping one another’s attitudes about what it means to succeed, struggle, and belong (Harris, 1995; Ryan, 2000). For example, when at-risk youth are grouped together in poorly structured interventions, peer dynamics can reinforce problem behaviors, highlighting how social contagion operates in unsupervised settings (Dishion & Dodge, 2005). In academic contexts, students who perceive their peer climate to be more positive and supportive tend to move away from demonstration-avoidance goals—aims to avoid looking incompetent or inferior in front of others (Makara & Madjar, 2015). Similarly, adolescents become more similar to their peers in math anxiety over time (Kim et al., 2023). Perhaps most relevant evidence is the recent work by Hemi et al. (2024), which found that students’ perceptions of their peers’ achievement goals were significantly associated with their own. This suggests that motivational states like achievement goals and anxiety are socially transmissible, shaped by the peer environment rather than individual differences alone.

We propose that one way peer mindset culture influences students’ sense of belonging is through this social contagion. Students interpret cues from those around them, especially peers, about what kind of beliefs are socially rewarded (Ryan, 2000). When those around them express fixed mindset ideas, even subtly, students may begin to internalize and adopt those beliefs (King, 2020), sometimes to fit in and avoid the discomfort of standing out. This pattern is empirically supported by a recent meta-analysis linking peers’ mindsets to students’ own mindsets (Fong et al., 2025b). This process can happen in everyday, seemingly harmless ways. A student who earns a top score on a math exam might say, “I barely studied. I guess I am good at this,” sending a signal to peers that ability is innate and effortless (Seo et al., 2025b). When such messages come from multiple directions, or from socially influential peers, even students who started with a growth mindset may begin to shift their beliefs (King, 2020) not only because fitting in feels more important than holding onto a countercultural view, but also because they may have been genuinely persuaded over time.

This kind of conformity may protect students from the discomfort of a mindset-culture mismatch in the short term. But we argue that over time, adopting a fixed mindset through social contagion can come at a significant cost, especially when students encounter challenges. If a student comes to believe that ability is fixed, and then later struggles in class, they may interpret that struggle as a sign that they do not belong, or that they are “not cut out for it” (Muenks et al., 2021). These beliefs can lead to decreased motivation, withdrawal from challenge, and eventually disengagement from the domain altogether (Wallace et al., 2023; Williams et al., 2022; Yeager & Dweck, 2012).

Of note, not all peer interactions lead to social contagion. We know from prior work that contagion depends on several factors. First, the process requires time and sustained exposure. In one study, King (2020) found that students’ mindsets began to align with those of their peers only after several months of consistent interaction, but not when the exposure was shorter in duration. Second, students are more likely to adopt beliefs from peers they feel close to (Radel et al., 2015), as emotional bonds increase the likelihood of internalization. Third, peer status matters. Socially prominent peers, those who attract attention and admiration, are especially influential in setting cultural norms (Laninga-Wijnen et al., 2018; Paluck et al., 2016). And finally, students with fewer social connections may be particularly susceptible, as they have fewer reference points and often look to their immediate peer group to guide their behavior (Faur et al., 2023).

Taken together, we argue that peer mindset culture can gradually shape students’ beliefs about ability, in part because fitting in often feels psychologically safer than maintaining a countercultural view. We believe that students with a weak or uncertain growth mindset may be particularly vulnerable, as their beliefs are more susceptible to social cues. Over time, if students come to adopt fixed mindset beliefs, these beliefs are likely to undermine their sense of belonging, especially when they begin to struggle academically (Williams et al., 2022).

### Pathway 2: Belonging Undermined by Social Disconnection

In this section, we examine how fixed mindset peer cultures can reduce students’ opportunities for connection, making it harder for them to feel supported, included, or seen by their peers. Although a sense of belonging can emerge from simply feeling that one’s important identities are respected, real, reciprocal relationships often play a critical role (Allen et al., 2021), especially during times of struggle. We propose that mindset-based peer selection, along with norms that discourage help-seeking, can fragment social networks and erode the foundation of peer support essential for belonging.

#### Example 1: Selection

As noted in the previous section, conforming to peer norms, such as adopting a fixed mindset, may carry long-term academic costs. But resisting those norms can also come with social costs due to peer selection. Peer selection refers to the tendency for students to form and maintain friendships with others who share similar attitudes, beliefs, or values. While peer influence through socialization is important, the process of selecting into like-minded peer groups is even more powerful in shaping students’ day-to-day social experience (Laninga-Wijnen & Veenstra, 2021). We argue that if students with fixed mindsets tend to gravitate toward peers who reinforce their worldview and are less inclined to engage with those who endorse growth mindset beliefs, this may contribute to the formation of homogenous peer cultures that reinforce prevailing mindset norms while excluding those who differ. For example, imagine a peer group that values innate talent. In such a setting, students who reject that norm and instead emphasize effort may be perceived as less capable, and peers may overtly or subtly exclude them from close relationships. While not all beliefs influence peer selection, we argue that mindset beliefs are likely to do so in educational settings, where views about learning and ability are particularly salient.

We propose that selective affiliation may be especially consequential for students from historically minoritized backgrounds. Recent evidence shows that Black and Latinx students reported lower belonging in classrooms where their Asian and White peers endorsed more fixed mindset beliefs (Lee et al., 2025), whereas the reverse was not true. This echoes prior research suggesting that minoritized students often enter academic spaces without an assumed sense of belonging and are more attuned to cues about inclusion (Murphy et al., 2007). Therefore, we argue that for students already facing structural inequalities, the effects of such peer disconnection are likely to be especially pronounced.

One might ask whether the opposite could also be true: Could students with a strong fixed mindset feel alienated in a growth mindset peer culture due to a similar selection process? In theory, yes; any mismatch between personal beliefs and peer norms could create friction. But we suggest that the effects are likely to be less severe in growth mindset peer cultures for one key reason: growth mindset cultures tend to be more inclusive. When peers value effort and view struggle as part of the learning process, they are more likely to offer support to one another and create space for second chances (Muenks & Yan, 2024; Seo et al., 2025b). In contrast, fixed mindset cultures often discourage help-seeking and may view failure as disqualifying (Freeman & Getty, 2023; Won & Chang, 2024). Thus, while students with fixed beliefs may initially feel out of sync in a growth mindset peer group, we argue that they are more likely to be met with psychological resources that can help them adapt.

#### Example 2: Limited Access to Social Support

Belonging does not require many friends, but it does require believing that support will be there when needed (Allen et al., 2018; Maluenda-Albornoz et al., 2023; Zhu et al., 2025). Students rely on peers not only for companionship but also for practical support. Academic help-seeking is especially important in learning environments where struggle is a normal part of development (Fong et al., 2021; Ryan & Pintrich, 1997). We propose that fixed mindset peer cultures may undermine belonging by discouraging both the giving and receiving of help (Muenks & Yan, 2024; Seo et al., 2025b), narrowing students’ opportunities to feel seen, supported, and connected.

Students who hold fixed mindsets often interpret struggle as a sign of low ability (Dweck, 1999). This belief not only makes them more hesitant to ask for help themselves, but it also alters how they perceive others who are struggling. If effort is seen as evidence of inadequacy, then classmates who are struggling may be viewed as “not cut out” for success. In this context, offering help may feel futile or even socially inappropriate (Rogers et al., 2023; Yu & McLellan, 2020). In addition, fixed mindset peer cultures often co-occur with performance goal orientations, as both emphasize proving competence and outperforming others rather than developing ability, leading students to focus on proving their ability rather than improving through effort (Dweck, 1986; Lee & Seo, 2019). In such environments, helping others can feel risky. Why support a peer who might compete with you for top rankings, awards, or praise? A zero-sum mentality can take root, reinforcing the idea that success is a scarce commodity to be protected, not shared (Chernyak-Hai & Davidai, 2022). This can lead to lower rates of peer support, not necessarily because students are unkind, but because they have internalized a belief system that status and recognition are limited resources. This motivational climate suppresses help-seeking as well (Ryan & Pintrich, 1997). When students fear that asking for help will be interpreted as incompetence, they are less likely to reach out even when they need it most.

Importantly, we suggest that perceptions of social support may be deeply intertwined with other mechanisms in our framework. For instance, when students believe they are being judged based on stereotypes, or when their values seem at odds with dominant mindset norms (as in mindset-culture mismatch), they may also come to perceive support as unavailable. Likewise, we argue that when students are excluded through mindset-based peer selection processes, what is lost is not only friendship but also access to the social support that peers can provide.

Although this mechanism may apply to all students, we believe it is especially consequential for students who are struggling, whether academically, emotionally, or socially. And for students from minoritized or underrepresented backgrounds, who may already face structural barriers to belonging, the absence of peer support can also be particularly costly (Becerra, 2012; Lett & Wright, 2003). For these students, supportive peer relationships are not just a nice-to-have; they are a critical source of affirmation that helps school feel like a place where they belong.

## Cultivating Belonging Through Peer Mindset Interventions

If peer mindset culture shapes students’ sense of belonging, interventions aimed at promoting belonging may need to address not just individual beliefs, but the broader peer environment. This raises a critical question: How can we shift classroom social norms so that peers collectively promote, rather than undermine, growth-oriented messages?

A common approach in mindset intervention research is to focus on the beliefs of individual students, helping them internalize the idea that abilities can grow with effort and support (e.g., Paunesku et al., 2015; Yeager et al., 2019). But changing individual beliefs may not be sufficient to transform peer culture. Believing something privately is not the same as expressing it publicly (Murphy et al., 2021). We have seen this limitation in other domains. Research on teacher mindset, for example, finds that what teachers believe and what students perceive can diverge significantly (Muenks et al., 2023). Instructors may report a strong growth mindset on surveys, but if they communicate those beliefs in ways that feel cold, controlling, or conditional, students may interpret them as fixed-minded (Murphy et al., 2021). In short, communication matters.

We propose that the same disconnect likely exists among peers. Students may believe in their own potential, but unless they convey that belief through behaviors, like normalizing struggle or modeling adaptive responses to failure, the culture may not shift. And in adolescence, where peer norms are especially salient, what gets said and seen often matters more than what is silently believed (for indirect evidence in other domains: Ajzen & Fishbein, 1977; Sandstrom & Bartini, 2010; Wicker, 1969). This suggests that cultivating a peer culture supportive of belonging requires more than distributing growth mindset materials or encouraging self-reflection. It requires intentional design of interventions that target public behaviors and perceived norms.

Although there are no interventions to date that explicitly target peer mindset culture, we see promising guidance from related work. Interventions that aim to clarify perceptions of peer norms and improve the communication of growth-oriented beliefs, especially by leveraging social dynamics like the need to belong or the influence of high-status peers, may hold particular promise for cultivating inclusive peer cultures (see Table 2). These strategies represent an important next step: not just helping students think differently about themselves but also reshaping how they perceive their peers and how they signal affirming beliefs to one another.

**Table 2:** Strategies to design peer mindset interventions to enhance belonging

**Target area**	**Target population**
Shifting individual students’ mindsets	Peer groups with members who predominantly hold a fixed mindset
Altering perceptions of peers’ mindsets	Students who are more susceptible to the interpretation of ambiguous social cues
Enhancing mindset-related behaviors	Peer groups with a growth mindset that lack effective communication skills
**Strategy for Change**	**Implementation Examples**
Leveraging the need to belong	Encouraging growth mindset-supportive peer groups or close peer relationships Targeting high social status peers (referred to as “social referent” peers) to establish growth-mindset norms
Consideration of broader context	Implementing interventions through teacher-led initiatives

### What to Target?

#### Individual Students’ Mindsets

In classrooms where fixed mindset beliefs are dominant, we propose that shaping peer culture may first require addressing individual beliefs. To reduce the risk of social contagion or peer selection processes reinforcing fixed mindset norms, it may be necessary to shift students’ beliefs about ability early on, helping them see the value and credibility of a growth mindset (Yeager et al., 2019). Importantly, the timing of these interventions matters. Research consistently shows that interventions are most impactful during key educational transitions, when students face uncertainty and are especially open to new ways of thinking (Blackwell et al., 2007; Yeager & Walton, 2011). We suggest that targeting mindset beliefs during these transitions may help create a stronger foundation for shaping peer culture moving forward.

#### Perceptions of Peers’ Mindset

Adolescents often operate in socially ambiguous environments (Graham, 2020). A classmate’s silence, competitiveness, or self-deprecating comment can be difficult to interpret, as it may reflect insecurity, confidence, or a fixed belief. Attribution theory reminds us that students are not passive observers; they actively construct meaning from others’ behavior, particularly in contexts that evoke uncertainty or stereotype threat (Graham, 2020). Notably, even subtle contextual factors, such as the ethnic composition of the classroom, can shape how students interpret ambiguous peer behaviors (Graham, 2018). In parallel, students may engage in what we call interpersonal mindset interpretation—inferring whether their peers view ability as something that can grow or as an unchangeable trait.

Drawing on motivational climate theory (Robinson, 2023) and the integrative framework of belonging (Allen et al., 2021), we propose that peer mindset culture includes both actual behaviors and students’ subjective interpretations (Fig. 1). Interventions, therefore, must address not just peer behaviors but how students make sense of them. Existing belonging interventions often focus on helping students reframe early academic struggles as normal and temporary (Walton & Brady, 2017; Walton & Cohen, 2007, 2011; Walton et al., 2015). We speculate that these interventions may work, in part, by altering how students interpret ambiguous peer signals and shaping their interpersonal mindset interpretations. For instance, students may misperceive the culture of their classroom due to pluralistic ignorance (Miller & McFarland, 1991), assuming others hold fixed beliefs when in fact many endorse growth mindsets but do not know how to communicate them. Even a single offhand comment like “I am not a math person” can lead others to incorrectly assume that fixed-mindset norms dominate the classroom (Campbell & Brauer, 2021). We suggest that guiding students to interpret such remarks not as definitive reflections of fixed beliefs but as fleeting expressions of frustration may help them view their peer norms as more open to growth.

In reality, most students report more growth-oriented beliefs than peers might expect (e.g., Dai & Cromley, 2014; Lee & Seo, 2019). And while fixed mindsets may persist in some academic fields (Leslie et al., 2015), we think that there are signs that norms are shifting, particularly in STEM, where inclusive teaching and mindset interventions are gaining traction (Education Week Research Center, 2016). Making these evolving norms visible can shift perceptions and, in turn, reduce identity threat and bolster belonging (Sparkman & Walton, 2017).

#### Mindset-Signaling Behaviors

If peer mindset culture is shaped by observable cues, then behavior becomes a critical point of intervention (Robinson, 2023). One reason is that even students who hold a growth mindset may inadvertently signal fixed beliefs, particularly in high-pressure environments where managing one's image is paramount (Niiya et al., 2010). Recent research has identified specific behaviors that convey peers’ implicit beliefs about ability. Muenks and Yan (2024) identified five such behaviors: competitiveness; self-deprecation (i.e., harshly critical self-evaluative statements in response to difficulty or failure, distinct from lighthearted jokes); fixed-oriented verbal messages (e.g., “I am a math person”); withdrawing effort when facing challenges; and showing disinterest in helping others. Seo et al. (2025) added two more: boasting about success and reluctance to share academic struggles.

Some behaviors signal a mindset explicitly. Fixed mindset beliefs are most overt in verbal messages like, “I am just not a math person,” which clearly communicate that intelligence is static (Muenks & Yan, 2024; Seo et al., 2025b). Similarly, students who boast about ease, such as claiming they performed well without studying or that an exam was “super easy,” can implicitly reinforce the notion that struggle is abnormal and that achievement is determined by fixed ability.

Others are more subtle but no less influential. When peers engage in harsh self-deprecation, describing themselves as “just not smart enough” or suggesting they have hit a personal ceiling, they may inadvertently signal a fixed mindset to others (Muenks & Yan, 2024). Likewise, when students refrain from sharing their struggles or difficulties, they may unintentionally reinforce the belief that competent students do not struggle (Seo et al., 2025b). For example, imagine a student sitting silently in a class where no one admits to being confused or needing help. In this environment, the absence of visible struggle can easily foster the illusion that everyone else finds the material easy. Over time, this silence may lead the student to infer that their peers believe ability is fixed and that success comes naturally to those who are "smart enough" (Seo et al., 2025b).

Our theoretical framework proposes that these mindset-signaling behaviors are key components of peer mindset cultures, and they shape peer mindset perceptions, which we argue influence students’ sense of belonging (Fig. 1). When students frequently hear fixed-mindset remarks, the environment can become threatening and socially isolating (Muenks et al., 2021; Seo et al., 2025b), especially for those struggling or navigating identity-based barriers (Burnette et al., 2013; Lee et al., 2025). Conversely, when students see peers openly share their challenges, support one another, and frame failure as part of the learning process, it sends a powerful signal: struggle is normal here, and failure facilitates learning and growth (Seo et al., 2025b). This belief about the meaning of struggles and challenges—sometimes referred to as a failure mindset—has been shown to promote adaptive learning behaviors, particularly when modeled socially (Haimovitz & Dweck, 2016). This insight echoes core findings from belonging interventions that normalize academic challenges as a shared and surmountable experience (Walton & Brady, 2022; Walton et al., 2023). The success of peer-led models, such as mentoring programs, is similarly rooted in this principle, demonstrating how students’ openness about struggle and growth can create more inclusive, affirming peer climates (Harrison et al., 2022; Hecht et al., 2022).

Importantly, these behaviors do not operate in isolation. In competitive classrooms, for example, students may be less willing to show vulnerability or offer help, amplifying the signals that reinforce a fixed mindset climate (Seo et al., 2025b). Yet the presence of one behavior (e.g., shared struggle) can buffer against the negative effects of another (e.g., competitiveness), illustrating the complex interplay of mindset-signaling cues that shape peer culture (Seo et al., 2025b). Ultimately, these social behaviors do more than shape perceptions; they shape the very possibilities for belonging in the classroom and in the field (Muenks et al., 2021; Seo et al., 2025b). Because they are both learnable and actionable, we argue that they offer a concrete and scalable target for peer-based interventions. By equipping students with the behavioral tools to express growth mindset values, we can shift the focus from individual belief change to broader cultural transformation, making classrooms more inclusive and fostering a stronger sense of belonging for all students.

### How to Change?

#### Leveraging Youth’s Motivation for Belonging and Status

Developmental research consistently shows that adolescents are highly attuned to their standing among peers (Albert et al., 2013) and that their behavior is often shaped by the need to be liked, respected, and included (Yeager et al., 2017). During this phase, belonging and social competence become intertwined (Yeager, 2024, Chapter 9). Youth learn to navigate peer norms not just to avoid rejection but to earn respect (Paluck & Shepherd, 2012). This makes adolescence an ideal time to promote prosocial norms (Bryan et al., 2016) if we can link them to admired identities and desirable peer roles (Chávez et al., 2025; Paluck et al., 2016).

One approach is to create environments where growth mindset behaviors, such as sharing struggles, helping peers, or framing effort positively, are not socially costly but instead socially rewarded, signaling acceptance and inclusion (Webel et al., 2010). Such environments matter because belonging and mindset culture operate as reciprocal contexts: when students, particularly first-generation students of color, feel accepted, respected, and included, they are more likely to put their growth mindset beliefs into practice, which in turn strengthens the link between those beliefs and academic performance (Fong et al., 2025a). In this sense, mindset culture both fosters belonging and is itself reinforced by belonging. One practical way to cultivate such environments is through peer discussion groups, small advisory circles, or dyadic support models (e.g., Smith et al., 2009). These smaller settings provide students with safer opportunities to express vulnerability and model growth mindset-oriented behaviors.

Another strategy draws from social referent theory: amplify the influence of high-status peers (Paluck & Shepherd, 2012). Adolescents tend to pay more attention to peers with high visibility or influence in their networks. By equipping these students with the tools and the motivation to model growth mindset behaviors, interventions can harness natural peer influence to shift broader norms (e.g., Chávez et al., 2025; Dijkstra et al., 2008; Paluck et al., 2016). Crucially, the goal is not to have high-status peers police others’ behavior, but to model inclusive, prosocial practices in authentic ways. When done well, students imitate, not because they are told to, but because they want to be associated with admired figures (Veenstra & Laninga-Wijnen, 2022).

### Aligning Peer Culture with the Broader Classroom Context

Peer-led change does not happen in isolation. One of the central lessons of the Mindset × Context framework is that psychological interventions are most effective when the surrounding culture supports their message (Carroll et al., 2023; Walton & Yeager, 2020). Research shows that even when students hold growth beliefs, their impact is limited if the broader context does not reinforce them (Hecht et al., 2023a, b; Yeager et al., 2022). Students may be open to adopting growth-oriented norms, but if the broader classroom structure incentivizes competition, scarcity, or comparison, peer behaviors that signal growth may be perceived as naïve or self-sabotaging.

Ames (1992) laid groundwork for understanding how classroom structures, such as evaluation methods, task design, and recognition practices, signal the value of effort versus ability and thereby shape the broader motivational climate. When course structures and policies enforce a grading curve or discourage student-driven group work, students are unlikely to feel safe asking for help. Similarly, when teachers frame success in a fixed way, such as rewarding only the top performers publicly without acknowledging effort or growth and suggesting that only some students can succeed, students may respond with fixed-mindset signaling behaviors, such as acting competitively and withholding help from others. When teachers frame failure in a fixed way, such as treating mistakes as evidence of low ability and failing to take responsibility for their own errors, students may also display fixed-mindset signaling behaviors, such as hiding their struggles rather than viewing them as part of the learning process. In this sense, peer mindset culture is shaped not just by students, but also by the practices, policies, and messages delivered by adults in the environment (Ames, 1992; Rattan et al., 2012). We believe that the implicit curriculum, or what students learn from how adults treat effort, mistakes, and collaboration, can reinforce or undermine peer norms.

This means that peer interventions should not be isolated. Teachers’ mindsets, grading practices, and classroom routines all shape how students behave (Laine & Tirri, 2023). As such, we propose that interventions that engage both teachers and students may create more durable change. For instance, teacher-led initiatives (e.g., Hecht et al., 2023a, b; Porter et al., 2022) can reduce structural pressures to compete and instead promote helping behaviors, creating a classroom environment that supports a peer culture grounded in collective growth.

Ultimately, we argue that the most promising interventions will support alignment across levels: individual mindsets, peer norms, and teaching practices. When teachers, peer, and students' own beliefs system all send coherent signals that learning is valued, effort is respected, and failure is part of growth, students receive a consistent message about what it means to belong and succeed, potentially maximizing the intervention’s impact.

## Toward the Future

In this paper, we proposed a theoretical model suggesting that peer mindset cultures may play a critical role in shaping students’ sense of belonging. Our argument extends the Mindset × Context framework by emphasizing that the peer group is not just a passive backdrop for learning but an active force that can either amplify or dampen students’ motivation and belonging. Fixed mindset peer cultures, we argue, may heighten stereotype threat and weaken social support, particularly for students from minoritized backgrounds. And for students who themselves hold growth mindsets, immersion in a fixed mindset culture may create a mismatch that leads to social exclusion.

What comes next is an empirical agenda. The mechanisms we outline—stereotype threat, mindset-culture mismatch, social contagion, peer selection, and low peer support—are currently theoretical and require rigorous testing to establish their validity and impact. These pathways are not necessarily equally potent, nor are they exhaustive. Future research should examine which of these mechanisms operate most strongly, under what conditions, and for whom. Clarifying these processes will not only refine our understanding of peer influence but will also help identify precise targets for intervention. Rather than broadly encouraging more positive peer cultures, we can begin to ask: Which aspects of peer interaction matter most for whom, and how can we design interventions that move the needle on those dimensions?

Beyond testing mechanisms, future studies should also take seriously the layered nature of educational environments. Mindset × Context theory (Hecht et al., 2021; Walton & Yeager, 2020) reminds us that psychological processes unfold within systems, which include not only peer cultures but also classroom norms, school-wide practices, and structural conditions. A growth mindset may be an asset for a struggling student, but its potential will be blunted if the peer culture discourages vulnerability, if teachers implicitly reward innate talent, or if the school lacks the supports needed to turn effort into improvement (Seo et al., 2025c). Understanding how various levels of psychological affordances interact with structural constraints is essential for making meaningful progress, not just in theory, but in practice (e.g., Covarrubias, 2023; Kroeper et al., 2025). The goal, then, is not merely to accumulate evidence about peer mindset cultures in isolation, but to understand how they function as one piece of a broader developmental ecology. As the field moves forward, research should strive to capture this complexity, linking micro-level social behaviors to macro-level outcomes, and designing interventions that acknowledge the full range of influences that shape students’ experiences of belonging and success.

## Conclusion

Despite decades of research on sense of belonging, we still lack a clear understanding of *how* peer mindset cultures shape students’ sense of belonging. This paper addresses that gap by introducing a theoretical framework that explains how peer mindset culture functions as a social force shaping students’ sense of belonging in school settings. We identify two psychological pathways—identity threat and social disconnection—through which fixed-mindset peer norms may undermine belonging, particularly for students from historically minoritized groups, those who are struggling academically, and those who personally endorse a growth mindset. Within these pathways, we outline five empirically testable mechanisms that explain how students interpret peer behaviors and form judgments about their social fit. Our framework contributes to belonging research by highlighting peer mindset culture as a key social context through which students interpret whether effort and growth are valued and, in turn, whether they belong . It also points to new directions for intervention: efforts to promote belonging must consider not only individual beliefs but also peer norms and their alignment with teacher practices. By attending to these social dynamics, we can better understand how belonging is developed through students’ interactions with their peers.
